# Hydrogen Sulfide Inhibits Formaldehyde-Induced Endoplasmic Reticulum Stress in PC12 Cells by Upregulation of SIRT-1

**DOI:** 10.1371/journal.pone.0089856

**Published:** 2014-02-28

**Authors:** Xiang Li, Kai-Yan Zhang, Ping Zhang, Li-Xun Chen, Li Wang, Ming Xie, Chun-Yan Wang, Xiao-Qing Tang

**Affiliations:** 1 Department of Anesthesiology, the First Affiliated Hospital, University of South China, Hengyang, Hunan, P. R. China; 2 Department of Neurology, the First Affiliated Hospital, University of South China, Hengyang, Hunan, P. R. China; 3 Institute of Neuroscience, Medical College, University of South China, Hengyang, Hunan, P. R. China; 4 Department of Neurology, Nanhua Affiliated Hospital, University of South China, Hengyang, Hunan, P. R. China; 5 Department of Anthropotomy, Medical College, University of South China, Hengyang, Hunan, P.R. China; 6 Department of Pathophysiology, Medical College, University of South China, Hengyang, Hunan, P.R. China; University of Louisville, United States of America

## Abstract

**Background:**

Formaldehyde (FA), a well-known environmental pollutant, has been classified as a neurotoxic molecule. Our recent data demonstrate that hydrogen sulfide (H_2_S), the third gaseous transmitter, has a protective effect on the neurotoxicity of FA. However, the exact mechanisms underlying this protection remain largely unknown. Endoplasmic reticulum (ER) stress has been implicated in the neurotoxicity of FA. Silent mating type information regulator 2 homolog 1 (SIRT-1), a histone deacetylases, has various biological activities, including the extension of lifespan, the modulation of ER stress, and the neuroprotective action.

**Objective:**

We hypothesize that the protection of H_2_S against FA-induced neurotoxicity involves in inhibiting ER stress by upregulation of SIRT-1. The present study attempted to investigate the protective effect of H_2_S on FA-induced ER stress in PC12 cells and the contribution of SIRT-1 to the protection of H_2_S against FA-induced injuries, including ER stress, cytotoxicity and apoptosis.

**Principal Findings:**

We found that exogenous application of sodium hydrosulfide (NaHS; an H_2_S donor) significantly attenuated FA-induced ER stress responses, including the upregulated levels of glucose-regulated protein 78, C/EBP homologous protein, and cleaved caspase-12 expression. We showed that NaHS upregulates the expression of SIRT-1 in PC12 cells. Moreover, the protective effects of H_2_S on FA-elicited ER stress, cytotoxicity and apoptosis were reversed by Sirtinol, a specific inhibitor of SIRT-1.

**Conclusion/Significance:**

These data indicate that H_2_S exerts its protection against the neurotoxicity of FA through overcoming ER stress via upregulation of SIRT-1. Our findings provide novel insights into the protective mechanisms of H_2_S against FA-induced neurotoxicity.

## Introduction

Formaldehyde (FA), a member of the aldehyde family and one of the simplest organic molecules, is a well-known indoor and outdoor pollutant [1]. The central nervous system (CNS) is one of the most important systems affected by FA and the neurotoxic effects of FA in the human health have attracted extensive studies. Epidemiological data showed that behavioral and neurological symptoms occur in histology technicians and workers exposed to high levels of FA over a long time [2], [3]. In several experimental models, it has been demonstrated that FA exposure induces the neurotoxicity and apoptosis in the cultured cortical neurons and PC12 cells in vitro [4]–[7] causes various morphological changes in the brain of rats [8]–[10], and elicits behavioral and learning and memory disorders in rats [11]–[14]. These findings confirm FA-induced neurotoxicity. Moreover, increasing evidence documents that the elevated endogenous FA levels contribute to the pathology of Alzheimer's disease [15]–[17]. Therefore, it is of utmost importance to develop new therapeutic approaches to prevent the neurotoxicity of FA.

Hydrogen sulfide (H_2_S), the third gaseous mediator alongside with nitric oxide and carbon monoxide [18]–[20], is recognized as a novel endogenous neuroprotectant [21]–[25]. Interestingly, our recent data demonstrate the protection of H_2_S against the neurotoxicity of FA [26], which for the first time implies a promising future of H_2_S-based preventions and therapies for neuronal damage induced by FA exposure. However, the exact mechanism of H_2_S-attenuated FA neurotoxicity remains largely unknown.

The endoplasmic reticulum (ER) is an important organelle responsible for the synthesis and folding of proteins that are required for cell survival and normal cellular functions [27]. Excessive and prolonged stress impairs ER function and leads to an accumulation of unfolded or misfoldedproteins, which induces ER stress [28]. Important roles for ER stress and ER stress-induced cell death have been reported in a broad spectrum of pathological conditions [27], [29]. Recently, Luo et al. reported that one of mechanisms of FA-induced neurotoxicity involves ER stress [6]. When PC12 cells are exposed to FA, the expressions of ER stress response genes, such as GRP78 (78-kDa glucose-regulated protein), CHOP (CEBP homology protein), and cleaved caspase 12 are up-regulated, which indicate that modulation of ER stress could represent a promising approach for prevention or treatment of FA neurotoxicity [6]. Considering that ER stress-induced apoptotic cell death is a critical step in the pathogenesis of FA neurotoxicity, and H_2_S can function as a survival factor for neurons, these findings prompted us to wonder whether H_2_S mediate its protective effect against FA-induced neurotoxicity by inhibiting the ER stress pathway.

Silent mating type information regulator 2 homolog 1 (SIRT-1), one of the nicotinamide adenine dinucleotide (NAD^+^)-dependent histone deacetylases, plays a critical role of in the longevity effects elicited by calorie restriction. Recently, accumulating evidence has shown that SIRT-1 is a neuroprotective molecule, which protects neurons against cellular damage and stressful perturbations in both acute and chronic neurological diseases [30]–[32]. It has been reported that SIRT-1 exerts its beneficial effects on insulin resistance via attenuating ER stress [33], [34]. Thus, we speculated that H_2_S inhibits FA-induced ER stress by regulation of SIRT-1.

The present study was to test the ability of H_2_S in reducing FA-induced ER stress and the mediatory role of SIRT-1 in this protective action of H_2_S. We for the first time demonstrated that H_2_S attenuates FA-induced ER stress in PC12 cells, which is involved in upregulation of SIRT-1.

## Materials and Methods

### 1. Materials

Sodium hydrosulfide (NaHS, a donor of H_2_S), formaldehyde, Sirtinol (the specific inhibitor of SIRT-1), Hoechst 33258, propidium iodide (PI), and RNase were purchased from Sigma Chemical CO (St.Louis, MO, USA). Cell counting kit-8 (CCK-8) was supplied by Dojindo Molecular Technologies, Inc. (Rockvile, MD, USA). Annexin V was purchased from Nanjing KeyGEN Biotech Co., Ltd. (Nanjing, China). Specific monoclonal anti-SIRT-1 antibody was obtained from Abcam (Hong Kong, China). Specific monoclonal antibodies to GRP78 and CHOP were purchased from Epitomic Inc (Burlingame, UK). Specific monoclonal anti-Caspase-12 antibody was obtained from Sigma Chemical (St Louis, MO, USA). RPMI-1640 medium, horse serum and fetal bovine serum were supplied by Gibico, BRL (Ground Island, NY, USA).

### 2. Cell culture

The PC12 cell line was derived from rat pheochromocytoma, a tumor arising of the adrenal medulla [35] and represents a valuable model to study cell fate such as neuronal differentiation, cell proliferation, or cell survival [36]–[38]. PC12 cells were (ATCC, CRL-1721) generously provided by Sun Yat-sen University Experimental Animal Center (Guangzhou, China) and grown in PMI-1640 medium supplemented with 10% heat-inactivated horse serum and 5% fetal bovine serum (FBS) at 37°C under an atmosphere of 5% CO_2_ and 95% air. The culture media was changed three times per week.

### 3. Determination of cell viability

The viability of PC12 cells was determined by CCK-8 assay. PC12 cells were cultured in 96-well plates at 37°C under an atmosphere of 5% CO_2_ and 95% air. When the cells were about 70% confluent, indicated conditioned-mediums were administered. At the end of treatments, 5 µl CCK-8 solutions were added into each well and then the plates were incubated for further 3 h in the incubator. Absorbance at 450 nm was measured with a microplate reader (Molecular Devices, Sunnyvale, CA, USA). Means of five wells optical density (OD) in the indicated groups were used to calculate the percentage of cell viability according to the formula below: cell viability (%)  =  OD treatment group/OD control group×100%.

### 4. Nuclear staining for assessment of apoptosis

Chromosomal condensation and morphological changes in the nucleus of PC12 cells were observed using the chromatin dye Hoechst 33258. The PC12 cells were fixed with 4% paraformaldehyde in 0.1 M phosphate buffered saline (PBS) for 10 min. After three rinses with PBS, the cells were stained with 5 mg/L Hoechst 33258 for 10 min. Slides were rinsed briefly with PBS, air dried, and then mounted in an anti-fluorescein fading medium (Perma Fluor, Immunon, PA, USA). Slides were visualized under a fluorescent microscope (BX50-FLA, Olympus, Tokyo, Japan). Viable cells displayed normal nuclear size and uniform fluorescence, whereas apoptotic cells showed condensed nuclei or nuclear condensations.

### 5. Flow cytometry analysis of apoptosis with PI and Annexin V double staining

Treated PC12 cells were digested with trypsin (2.5 g/L) and collected in an Eppendorf tube. Cells were washed twice with PBS by centrifugation and then the supernatants were discarded. To detect apoptosis, 500 µl PBS, 5 µl Annexin V-FITC and 5 µl PI were added to each tube, and the contents of the tube were mixed in the dark at room temperature for 15 min, followed by FCM flow cytometric (FCM, Beckman-Coulter, Miami, FL, USA) testing. Cell Quest software (Becton-Dickinson) was used to acquire and analyze the data and the data are expressed as cell percentages.

### 6. Western blot analysis

PC12 cells were lysed in an ice-cold lysis buffer [20 mM Tris–HCl, pH 7.5, 150 mM NaCl, 1% Triton X-100, 1 mM phenylmethylsulphonylfluoride (PMSF), 1 mM Na_3_VO_4_, leupeptin, and EDTA]. And then the samples were centrifuged at 14 000 r.p.m. for 30 min at 4°C and the supernatant were obtained. Protein concentration was assessed using a BCA Protein Assay Kit (Beyotime, Shanghai, China). Equivalent amounts of protein for each sample were run on sodium dodecyl sulfate-polyacrylamide gel electrophoresis (SDS-PAGE). The proteins were then transferred to a PVDF membrane, and blocked in TBS-T buffer (50 mM Tris-HCl, pH 7.5, 150 mM NaCl, 0.05% Tween-20) containing 5% bovine serum albumin for 2 h. The membranes were incubated with blocking solution containing primary antibody (anti-CHOP, 1∶500; anti-GRP78, 1∶2000; anti-Caspase12, 1∶2000; anti-SIRT-1, 1∶2000) overnight at 4°C. After washing 3 times, the membrane was incubated in anti-rabbit secondary antibody conjugated to horseradish peroxidase (1∶5000) in blocking solution for 2 h. Next, the membrane was washed in TBS-T buffer and the electrogenerated chemiluminescence reaction solutions were added for 2 min. The signal of the immunoblots was visualized using an image analysis system equipped with a software BIO-ID (Vilber Lourmat, France).

### 7. Statistical analysis

Data are expressed as mean ± S.E.M. The significance of inter-group differences was evaluated by one-way analyses of variance (ANOVA: Least-significant difference test). Differences were considered significant at two tailed *P*<0.05.

## Results

### 1. FA triggers ER stress in PC12 cells

**Figure 1:**
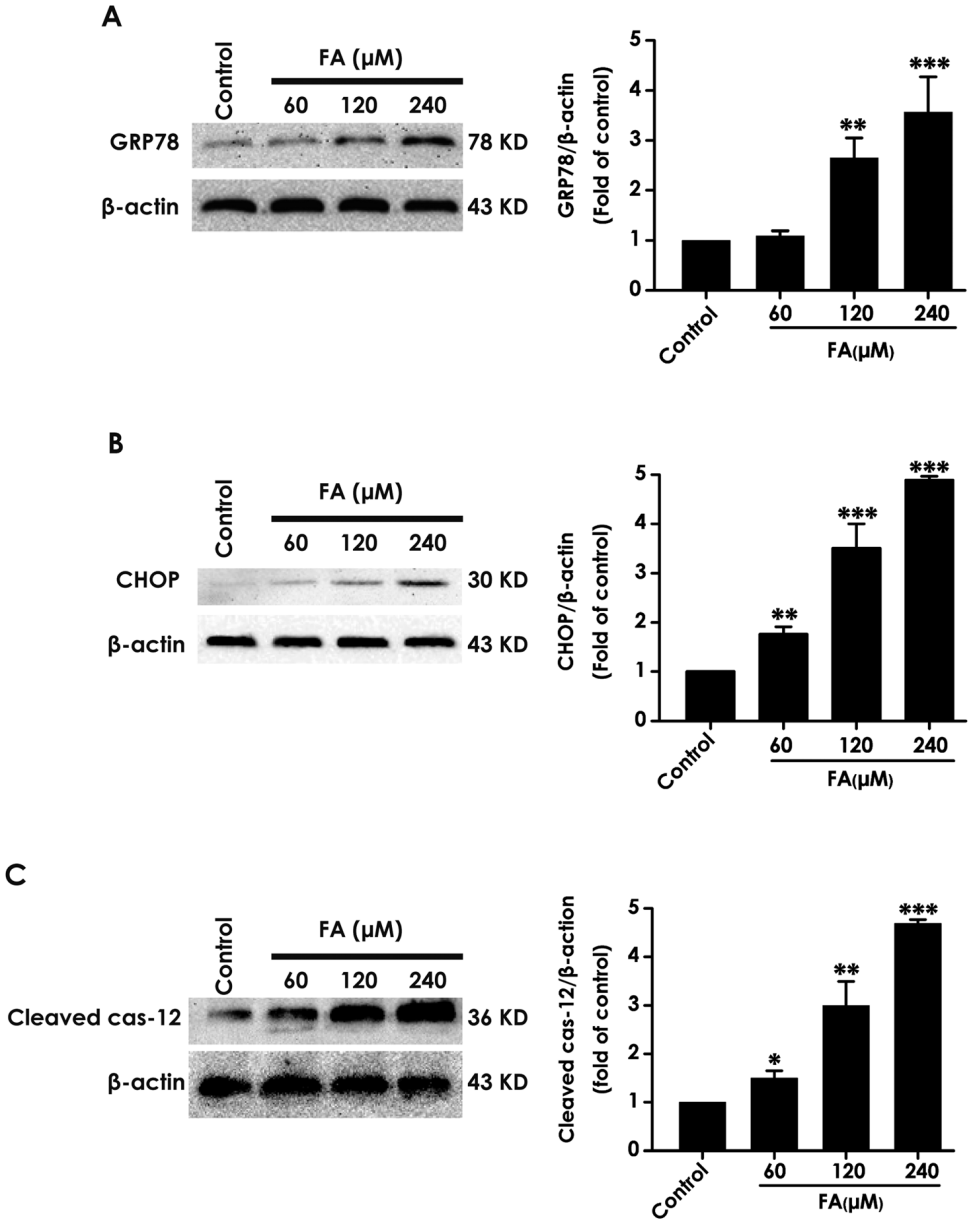
Effect of formaldehyde on the expressions of GRP78, CHOP and cleaved caspase-12 in PC12 cells. PC12 cells were exposed to different concentrations of formaldehyde (FA, 60, 120 and 240 µmol/L) for 24 h. The levels of GRP78 (A), CHOP (B), and cleaved caspase-12 (C) expression in PC12 cells were detected by Western blot using anti-GRP78, -CHOP, and -cleaved caspase-12 antibody, respectively. In all blots, staining for β-actin was used as a loading control. Values are the mean ± S.E.M. of three independent experiments. *P<0.05; **P<0.01; ***P<0.001, *vs* control group.

To explore whether the mechanism of the protective action of H_2_S against the neurotoxicity of FA involves regulation of ER stress, we first sought to investigate whether FA induces ER stress by measuring the expression levels of GRP78, CHOP, and cleaved caspase-12 in FA-treated PC12 cells using Western blot analysis. As illustrated in Fig. 1, after 24 h exposure of FA (60, 120, or 240 µmol), the amounts of GRP78, CHOP, and cleaved caspase-12 in PC12 cells were significantly increased. These data indicated that ER stress participates in FA-induced neurotoxicity.

### 2. H_2_S prevents FA-induced ER stress in PC12 cells

**Figure 2:**
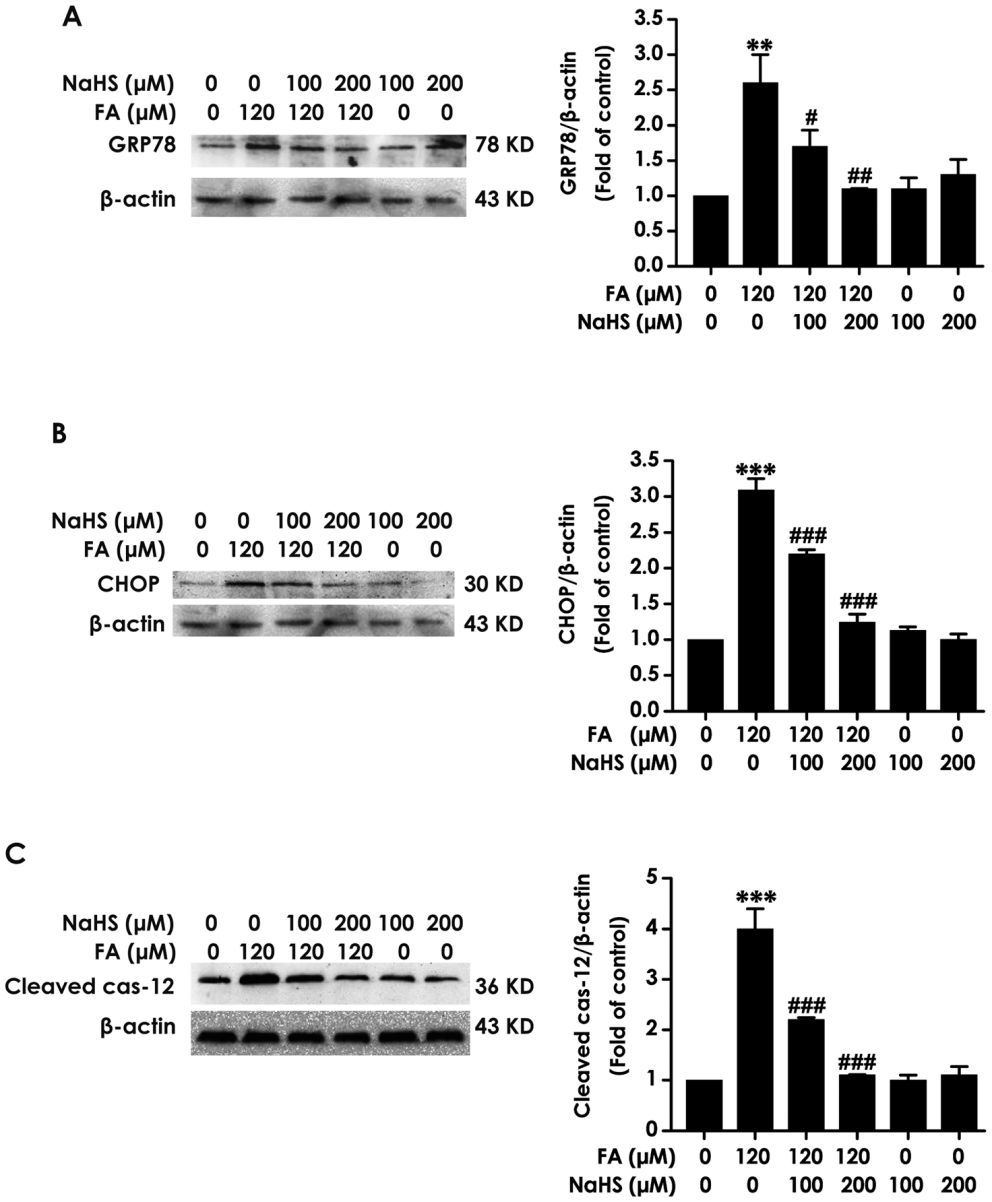
Effect of H_2_S on formaldehyde-induced ER stress in PC12 cells. After pretreated with NaHS at concentrations of 100 and 200 µmol/L for 30 min, PC12 cells were exposed to formaldehyde (FA, 120 µmol/L) for 24 h. The levels of GRP78 (A), CHOP (B), and cleaved caspase-12 (C) expression in PC12 cells were detected by Western blot using anti-GRP78, -CHOP, and -cleaved caspase-12 antibody, respectively. In all blots, staining for β-actin was used as a loading control. Values are the mean ± S.E.M. of three independent experiments. **P<0.01; ***P<0.001, *vs* control group; ^#^ *P*<0.05, ^##^ *P*<0.01, ^###^ *P*<0.001, *vs* FA-treated alone group.

Next, we investigated the effect of H_2_S on FA-induced ER stress in PC12 cells. As illustrated in Fig. 2, pretreatment with NaHS (100 or 200 µM) for 30 min significantly attenuated the increases in the expression levels of GRP78, CHOP, and cleaved caspase-12 in PC12 cells induced by treatment with 120 µM of FA for 24 h. These data indicated that H_2_S produces a protective effect against FA-induced ER stress.

### 3. H_2_S upregulates SIRT-1 expression in PC12 cells

**Figure 3:**
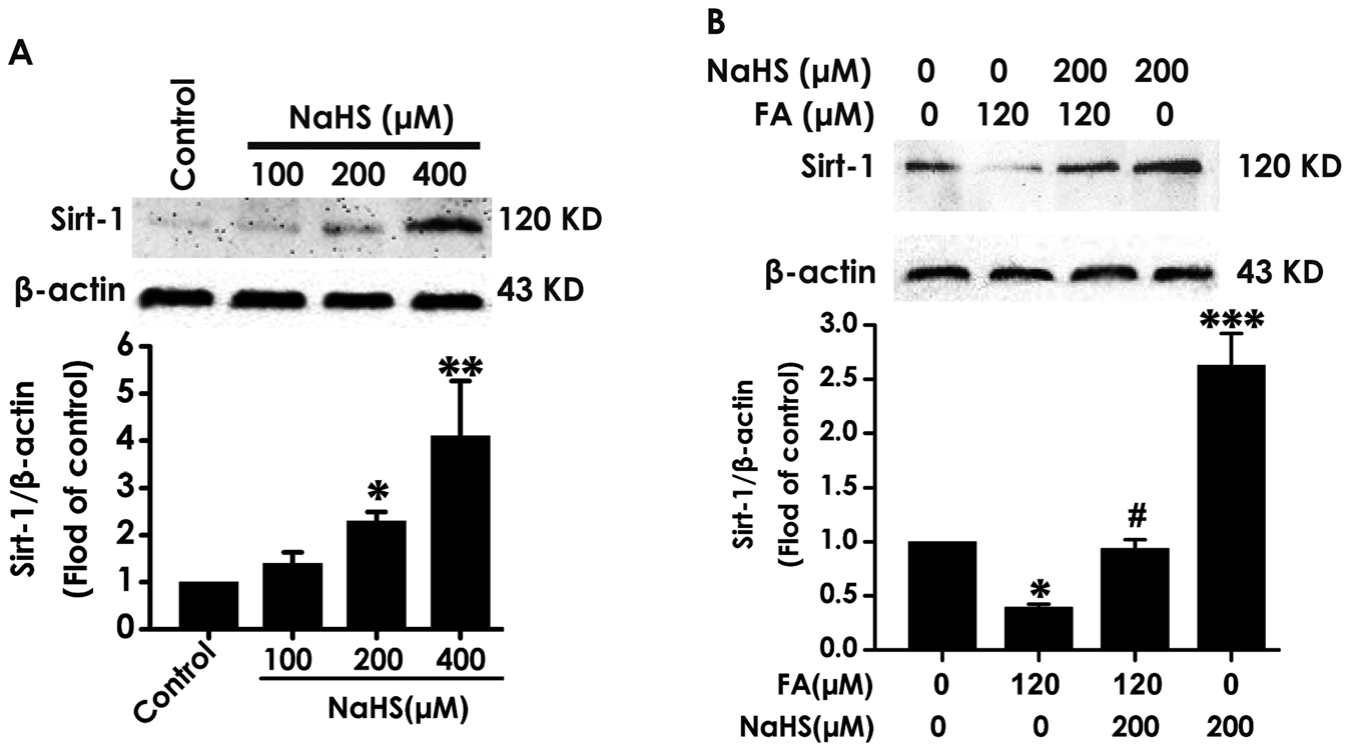
Effect of H_2_S on the expression of SIRT-1 in PC12 cells. A, PC12 cells were treated with different concentrations of NaHS (100, 200 and 400 µmol/L) for 24 h. B, PC12 cells pretreated with NaHS at a concentration of 200 µM for 30 min prior to 24-h exposure of formaldehyde (FA, 120 µM). The levels of SIRT-1 expression in PC12 cells were detected by Western blot using anti-SIRT-1 antibody. In all blots, staining for β-actin was used as a loading control. Values are the mean ± S.E.M. of three independent experiments. *P<0.05; **P<0.01; ***P<0.001, *vs* control group; ^#^ *P*<0.05, *vs* FA-treated alone group.

To explore whether SIRT-1 mediates the mechanism underlying the protective effect of H_2_S against FA-elicited ER stress, we first determined whether H_2_S regulates the expressions of SIRT-1 in PC12 cells. After treatment with different concentrations of NaHS (100, 200, and 400 µM) for 24 h, the expression of SIRT-1 in PC12 cells was markedly increased in a dose dependent manner (Fig. 3A). Furthermore, the inhibited SIRT-1 expression by exposure of FA (120 µM, 24 h) was significantly reversed by treatment with 200 µM of NaHS (Fig. 3B). These data indicate the up-regulatory role of H_2_S in SIRT-1 expression.

### 4. Inhibition of SIRT-1 reverses the protective effect of H_2_S against FA-induced ER stress in PC12 cells

**Figure 4:**
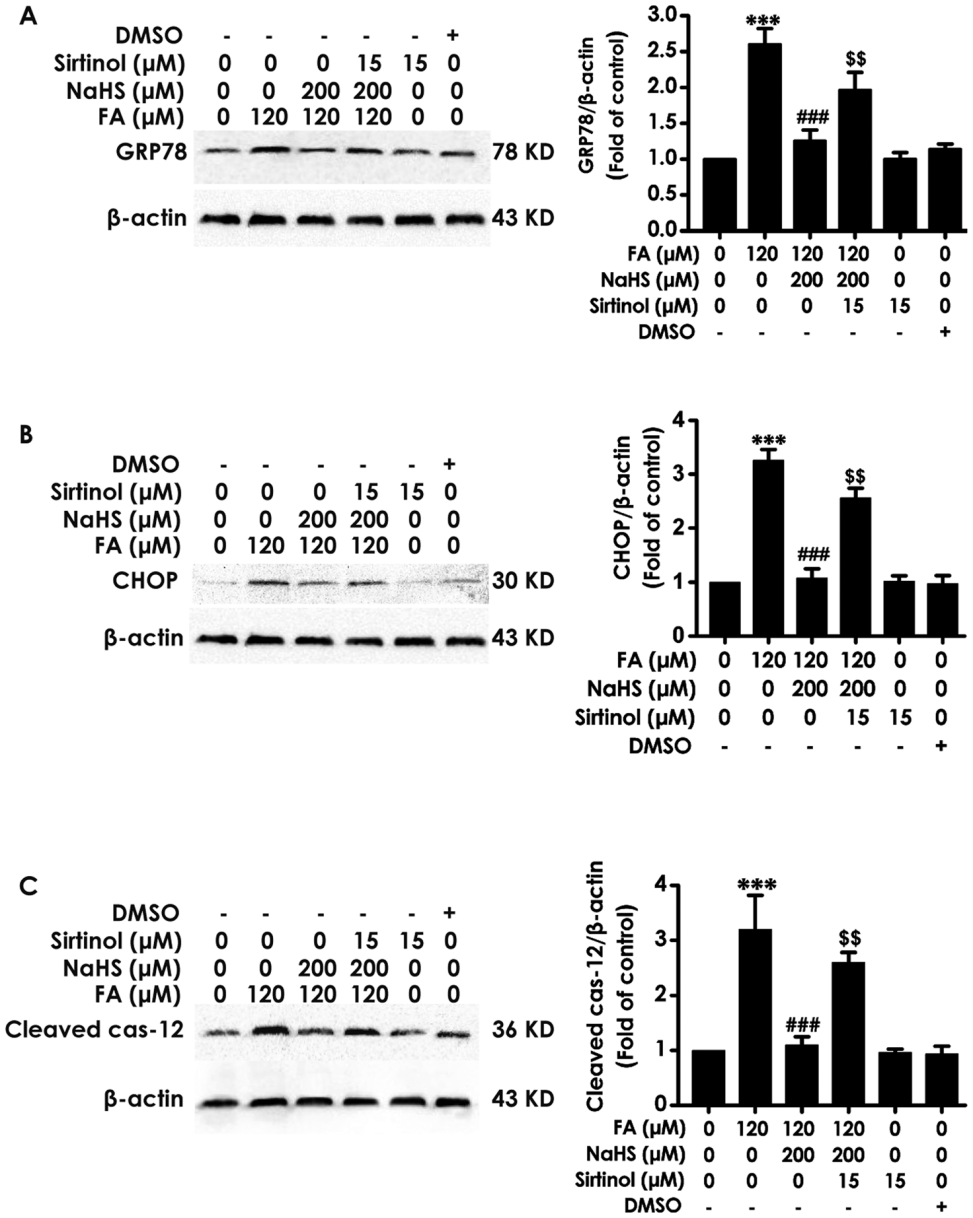
Effect of Sirtinol, a specific SIRT-1 inhibitor, on H_2_S-exerted protection against ER stress induced by formaldehyde. PC12 cells preincubated with Sirtinol (15 µM) 30 min before pretreatment with NaHS (200 µM) for 30 min prior to 24-h exposure of formaldehyde (FA, 120 µM). The levels of GRP78 (A), CHOP (B), and cleaved caspase-12 (C) expression in PC12 cells were detected by Western blot using anti-GRP78, -CHOP, and -cleaved caspase-12 antibody, respectively. In all blots, staining for β-actin was used as a loading control. Values are the mean ± S.E.M. of three independent experiments. ***P<0.001, *vs* control group; ^###^ *P*<0.001, *vs* FA-treated alone group; ^$$^ *P*<0.01, *vs* cotreated with NaHS and FA group.

To determine whether SIRT-1 mediate the protective effect of H_2_S on FA-induced ER stress in PC12 cells, we further explored whether Sirtinol, a specific inhibitor of SIRT-1, prevents this protection of H_2_S against ER stress. We pretreated PC12 cells with Sirtinol (15 µM) 30 min before the administration of NaHS (200 µM). As showed in Fig. 4, Sirtinol, the inhibitor of SIRT-1, significantly reversed the expression levels of GRP78, CHOP, and cleaved caspase-12 suppressed by NaHS, indicating that inhibition of SIRT-1 reverses the protective role of H_2_S against FA-induced ER stress. Sirtinol (15 µM) alone did not affect the expressions of GRP78, CHOP, and cleaved caspase-12 in PC12 cells. Taken together, these data suggest that SIRT-1 mediates the protective action of H_2_S against FA-induced ER stress.

### 5. Inhibition of SIRT-1 attenuates the protective effect of H_2_S against FA-induced cytotoxicity and apoptosis in PC12 cells

**Figure 5:**
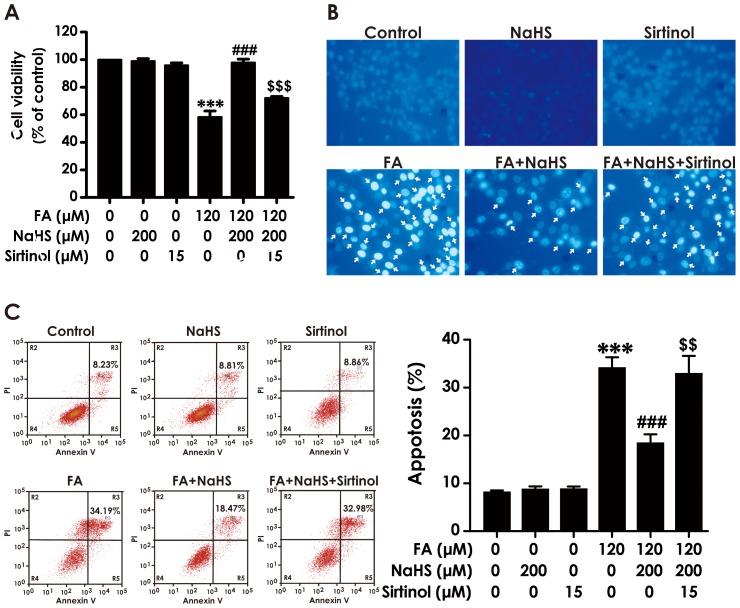
Effect of Sirtinol, a specific SIRT-1 inhibitor, on H_2_S-exerted protection against cytotoxicity and apoptosis induced by formaldehyde. PC12 cells preincubated with Sirtinol (15 µM) 30 min before pretreatment with NaHS (200 µM) for 30 min prior to 24-h exposure of formaldehyde (FA, 120 µM). A, The cell viability was determined by CCK-8 assay. B, The apoptosis of PC12 cells was visualized under a fluorescence microscope (10× objective, BX50-FLA, Olympus) after incubated with 5 mg/L Hoechst 33258 for 30 min. C, The apoptosis of PC12 cells was assessed by flow cytometry after PI and Annexin V double staining (The annexin-V^−^/PI^−^ population is made up of normal healthy cells, while annexin-V^+^/PI^−^cells exist in early apoptotic stage, and annexin-V^+^/PI^+^ cells exist in late apoptotic stage). Values are the mean ± S.E.M. of five independent experiments. ***P<0.001, *vs* control group; ^###^ *P*<0.001, *vs* FA-treated alone group; ^$$^ *P*<0.01, ^$$$^ *P*<0.001, *vs* cotreated with NaHS and FA group.

We further explored whether inhibition of SIRT-1 by Sirtinol reverses the protection of H_2_S against FA-induced cytotoxicity and apoptosis in PC12 cells. As shown in Fig. 5, pretreatment with Sirtinol (15 µM) significantly reversed NaHS (200 µM)-suppressed the loss of cell viability and the increase in apoptosis induced by FA (120 µM, 24 h), indicating that inhibition of SIRT-1 reverses the protective role of H_2_S against FA-induced ER stress-mediated neurotoxicity. Neither NaHS (200 µM) nor Sirtinol (15 µM) alone affected the cell viability and apoptosis of PC12 cells.

## Discussion

We have previously demonstrated the protective effect of H_2_S against the neurotoxicity of FA [26]. Abnormal ER stress contributes substantially to FA-induced neurotoxicity. Thus, modulation of ER stress becomes crucial in understanding the mechanisms of H_2_S-exerted protection against the neurotoxicity of FA. The present study was designed to investigate the role of H_2_S in regulating FA-induced neuronal ER stress and the underlying mechanisms. The main findings of the present work were the following: (1) H_2_S significantly overcomes FA-induced ER stress responses; (2) H_2_S upregulates the expression of SIRT-1 in PC12 cells; (3) Inhibition of SIRT-1 by Sirtinol reverses H_2_S-provided neuroprotective effects on FA-induced cytotoxicity, apoptosis, and ER stress. These results implicate the contribution of inhibiting ER stress to the protection of H_2_S against FA-induced neurotoxicity and suggest that the protection of H_2_S is involved in upregulation of SIRT-1.

Increasing evidence demonstrates the toxic effects of FA on central nervous system [3], [5]–[7], [12], [14]. It is crucial to develop effective therapeutic drugs and strategies that can reverse or alleviate the neurotoxicity. In our previous study, the neurotoxicity of FA has shown to be suppressed by H_2_S [26]. However, the exact mechanism underlying this protective role of H_2_S needs to be further studied. It has been shown that the neurotoxicity of FA is contributed to excessive ER stress. Therefore, regulation of ER stress becomes critical in understanding the contribution of H_2_S to the protection against FA neurotoxicity. In the present work, we examined the influence of H_2_S in the upregulatory effect of FA on the expression of protein markers of ER stress, such as GRP78, CHOP, and cleaved caspase-12, in PC12 cells. We demonstrated that administration of NaHS (the donor of H_2_S) alleviates the upregulated expressions of GRP78, CHOP, and cleaved caspase-12 in PC 12 cells exposed to FA. These results indicate that H_2_S is able to downregulate the elevated ER stress by FA. GRP78, an ER-chaperon protein, plays a crucial role in regulation of the ER dynamic homeostasis, and is a marker for ER stress [39]–[41]. CHOP, ubiquitously expressed at very low levels in normal cells, is upregulated in the presence of excessive ER stress. The increase of CHOP is a good biomarker of the presence of ER stress [42]. Pro-caspase-12 is localized on the cytoplasmic side of ER and proteolytically activated during excess ER stress. It has been reported that ER stress-induced apoptosis is mediated by the activation of CHOP and caspase-12 [43]–[45]. Our previous confirmed that H_2_S has the protective role against FA-induced cytotoxicity and apoptosis [26]. Thus, our results indicate that H_2_S-attenuated ER stress plays an important role in its protective effects against FA-induced cytotoxicity and apoptosis. Recent studies have demonstrated that H_2_S-suppressed ER stress contributes to its protective effect on atherosclerotic lesions [46], cardiotoxicity of doxorubicin [47], homocysteine-induced cardiomyocytic injury [48], and 6-hydroxydopamine-induced neurotoxicity [49]. These previous findings provide a reasonable explanation for our results. It revealed that H_2_S confers protection against FA-induced neurotoxicity through inhibition of ER stress. Since exposure of FA cause neurotoxicity [3], [5]–[7], [12], [14] and elevated endogenous FA levels contribute to the pathology of Alzheimer's disease [15]–[17], our study suggests a promising future of H_2_S-based preventions and therapies for FA-exerted neuronal damage.

We further investigated the possible mechanisms for the protective effect of H_2_S against FA-induced ER stress. SIRT-1, the best-characterized SIRT family member, regulates longevity in several model organisms and is involved in cell survival, differentiation, and metabolism [50]. Recent studies indicate that SIRT-1 functions as a neuroprotective agent and rescues neurons in both acute and chronic neurological diseases [30]–[32]. Furthermore, Sirt-1 has been identified as a defender of ER stress [33], [34]. Therefore, we focused on whether SIRT-1 mediates the protective role of H_2_S against FA-induced ER stress and neurotoxicity. We found that NaHS not only upregulated the expression of SIRT-1 in PC12 cells, but also reversed FA-induced downregulation of SIRT-1 in PC12 cells. Furthermore, our present data demonstrated that the inhibitor of SIRT-1 reversed the inhibitory effect of H_2_S on the expression of GRP78, CHOP, and cleaved caspase-12 in PC12 cells treated with FA. Our results suggest that H_2_S-exerted protection against FA-induced ER stress is involved in upregulation of SIRT-1. In addition, we showed that the inhibitor of SIRT-1 also reversed the protective action of H_2_S against FA-induced cytotoxicity and apoptosis. Taken together, our data imply that SIRT-1 mediates the protective role of H_2_S against FA-induced ER stress and neurotoxicity. However, further studies are needed to uncover the mechanisms of the upregulation of SIRT-1 by H_2_S. It has been reported that sulfhydration mediates various physiologic action of H_2_S [51]–[54]. In the future, we will focus on elucidating whether H_2_S modulates SIRT-1 by sulfhydration.

In conclusion, our present study demonstrates that H_2_S can overcome FA-induced ER stress and neurotoxicity in PC12 cells. H_2_S up-regulates SIRT-1 expression in PC12 cells. Moreover, inhibition of SIRT-1 reversed H2S-elicited protection against the ER stress and neurotoxicity induced by FA. These findings suggest that the protection of H_2_S against FA-induced neurotoxicity is involved in its inhibitory role in ER stress, which is mediated by SIRT-1. Our results provide important insights into the molecular mechanism underlying H_2_S-mediated protective role in the neurotoxicity of FA.
